# Comparison of femtosecond laser-assisted cataract surgery and conventional phacoemulsification on corneal impact: A meta-analysis and systematic review

**DOI:** 10.1371/journal.pone.0284181

**Published:** 2023-04-14

**Authors:** Hanle Wang, Xinyi Chen, Jingjie Xu, Ke Yao

**Affiliations:** Eye Center of the Second Affiliated Hospital, Medical College of Zhejiang University, Hangzhou, China; University of Warmia, POLAND

## Abstract

This meta-analysis aims to compare corneal injuries and function after femtosecond laser-assisted cataract surgery (FLACS) and conventional phacoemulsification surgery (CPS). A comprehensive literature search of PubMed, EMBASE, and the Cochrane Controlled Trials Register was conducted to identify randomized controlled trials (RCT) and high-quality prospective comparative cohort studies comparing FLACS with CPS. Endothelial cell loss percentage (ECL%), central corneal thickness (CCT), endothelial cell density (ECD), endothelial cell loss (ECL), percentage of the hexagonal cell (6A), and coefficient of variance (CoV) were used as an indicator of corneal injury and function. Totally 42 trials (23 RCTs and 19 prospective cohort studies), including 3916 eyes, underwent FLACS, and a total of 3736 eyes underwent CPS. ECL% is significantly lower in the FLACS group at 1–3 days (P = 0.005), 1 week (P = 0.004), 1 month (P<0.0001), 3 months (P = 0.001), and 6 months (P = 0.004) after surgery compared to CPS. ECD and ECL appeared no statistically significant difference between the two groups, except for the significant reduction of ECD at 3 months in the CPS group (P = 0.002). CCT was significantly lower in the FLACS group at 1 week (P = 0.05) and 1 month (P = 0.002) early postoperatively. While at 1–3 days (P = 0.50), 3 months (P = 0.18), and 6 months (P = 0.11), there was no difference between the FLACS group and the CPS group. No significant difference was found in the percentage of hexagonal cells and the coefficient of variance. FLACS, compared with CPS, reduces corneal injury in the early postoperative period. Corneal edema recovered faster in the FLACS group in the early postoperative period. In addition, FLACS may be a better option for patients with corneal dysfunction.

## Introduction

Cataract is one of the most common eye diseases and the major cause of vision loss worldwide [1]. Surgically removing the opacity lens and replacing it with an intraocular lens is currently the only treatment for cataracts [2]. Since its invention in the 1960s, phacoemulsification has continued to improve and remained the best therapy for cataracts [3]. Femtoseconds were first applied to promote the key steps of phacoemulsification in 2008 [4], such as corneal incision, lens fragmentation, and anterior capsulotomy [5]. Ever since the discussion on the comparison of femtosecond-laser assisted cataract surgery (FLACS) with conventional phacoemulsification surgery (CPS) has never ceased. Many studies these years have suggested the optimization of FLACS for cataract surgery, including enhancing the circularity of capsulotomy [6], reducing the effective phacoemulsification time (EPT) [7], and providing better IOL placement [8].

The transparency and barrier function of the cornea is mainly sustained by the corneal endothelium [9], composed of a single layer of the endothelial cell. The corneal endothelium has no regenerative capacity [10]. Once suffering from an injury, endothelial cells cannot proliferate [11], and the loss of endothelial cells is irreversible. The healing procedures occurred as the remaining surrounding endothelial cells enlarged and migrated to cover the damaged area [12]. As a result, the endothelial cells will increase in size and alter in shape from hexagonal to pleomorphic [13]. This leads to a change in the percentage of hexagonal cells (6A) and coefficient of variance (CoV), which illustrate the function of the residual endothelial cells. During cataract surgery procedures, phacoemulsification may increase the risk of endothelial cell loss [9]. It has long been shown that phacoemulsification results in approximately 4%-25% of endothelial cell loss [14, 15]. The negative effect of phaco cataract surgery on endothelium is multifactorial and largely due to thermal [16] and mechanical injury [17]. It has been proven to be associated with surgical instruments, phacoemulsification time, ultrasound energy, and contact with lens fragments during surgery [17–19]. Since femtosecond lasers are thought to modify the surgery procedure and lessen the usage of ultrasound, the effect of this technique on the endothelium is of concern.

The corneal indicators used in previous meta-studies were inadequate, and the included articles were not rigorous enough. A recently published meta-analysis selected endothelial cell loss (ECL) and central corneal thickness (CCT) as indicators to evaluate corneal damage. Kolb, et al., in the meta-analysis, noted a significant ECL decline in the FLACS group 1–3 months postoperatively, while there was no significant difference within 1 week and over 6 months. CCT was significantly higher in the CPS group in the early time. Later in the 6 months, the difference decrease [20]. It was worth mentioning that this paper not only included prospective but also retrospective studies, which are not as reliable as prospective studies and may lead to greater bias. In contrast, Chen et al. proposed in 2021 [21] that ECL was consistently significantly lower than CPS in the FLACS group in the first week after surgery. The study analyzed RCTs only, but there were multiple mistakes in the inclusion of the article. So, we supposed it is not credible enough. Besides ECL and CCT, there are other corneal indicators. As early as 2016, Chen et al. [22] used the endothelial cell loss rate (ECL%) to measure the damage to the corneal endothelium and concluded that the difference persists after surgery from 1 week to 3 months. ECL% is the ratio of the number of endothelial cells loss to preoperative endothelial cells, which eliminates the difference from baseline and therefore may be more statistically significant. However, the sample size was small at the time, and new related studies have been published in recent years. In addition, the morphology of the remaining corneal endothelial cells was also of our interest. It is represented by 6A and CoV, indicating the function of the residual endothelial cells. Corneal injury is an important effect of cataract surgery and is closely related to postoperative visual quality. Former studies had only discussed 1–2 corneal indicators. In our study, a variety of corneal indicators were selected to comprehensively evaluate the postoperative corneal condition. This meta-analysis aimed to compare corneal impact and function after FLACS and CPS to provide a reference for clinical application.

## Materials and methods

### Search strategy and inclusion criteria

The study followed the PRISMA guidelines (Preferred Reporting Item for Systematic Reviews and Meta-Analysis). PubMed, EMBASE, and Cochrane Library were searched by keywords: “femtosecond” OR “Femtolaser” AND “cataract” in full text. Complete and published clinical prospective trials comparing FLACS and CPS up to date December 31, 2021, were included. Reviews, conference abstracts, case reports, letters, correspondence articles, and editorials were excluded. The researches that combined with other ophthalmic surgery were excluded. Involved studies should meet the criteria as follow: 1) prospective randomized control trials or high-quality comparative cohort studies; 2) published in English or Chinese; 3) compared clinical indicators of patients undergoing simple cataract surgery with and without femtosecond laser assistant; 4) contains at least one indicator of ECD, ECL, ECL%, CCT, CoV, 6A.

### Screening process

Studies screening were carried out by two authors (HL. W and JJ. X) independently. Titles and abstracts were read to screen for qualified studies, and full-text reading was performed when necessary to determine eligibility for inclusion criteria. Articles in disagreement were confirmed by a third author (XY. C) after discussion.

### Quality assessment

The cohort studies were assessed by Newcastle-Ottawa Scale (NOS) [23]. The NOS is an 8-stars scale based on patient selection (four stars), comparability (one star), and outcomes (three stars). Cochrane Collaboration’s tool for risk of bias [24] was applied to evaluate the quality of included RCTs by two independent authors (HL. W, JJ. X), which had random sequence generation, allocation concealment, blinding of participants and personnel, blinding of outcome assessment, incomplete outcome data, selective reporting, and other biases.

### Data extraction and outcome measurements

A standard data form was used in the extraction process, including the basic information such as title, authors, years, experimental design, sample size, clinical indicators, etc. All the disagreements were discussed and solved before data analysis and all of the data was double-checked by a second reviewer. Corneal endothelium-related clinical indicators at different postoperative time points were recorded with mean and standard deviation. When standard deviation was not reported, data were ruled out. Data expressed as medians and quartiles were converted to mean and standard deviation by Luo’s formula [25]. Data containing subgroups in FLACS or CPS were combined.

### Data analysis

RevMan software (version 5.4; Cochrane Collaboration, Oxford, United Kingdom) was used in statistical analyses. The corneal indicators were recorded in continuous data presented by weighted mean differences (WMDs) with 95% CIs. Statistical heterogeneity was calculated using the chi-square test and I2 statistics, with I2 measures more than 50% being attributed to strong heterogeneity. When heterogeneity was demonstrated, random-effects models were used, otherwise fixed-effect models. It was regarded as a statistically significant difference between FLACS and CPS when the P value was less than 0.05. Sensitivity analysis assessed how the results would have changed if a single study had been omitted by a single-study deletion analysis.

## Results

### Literature research and trails characteristics

**Fig 1:**
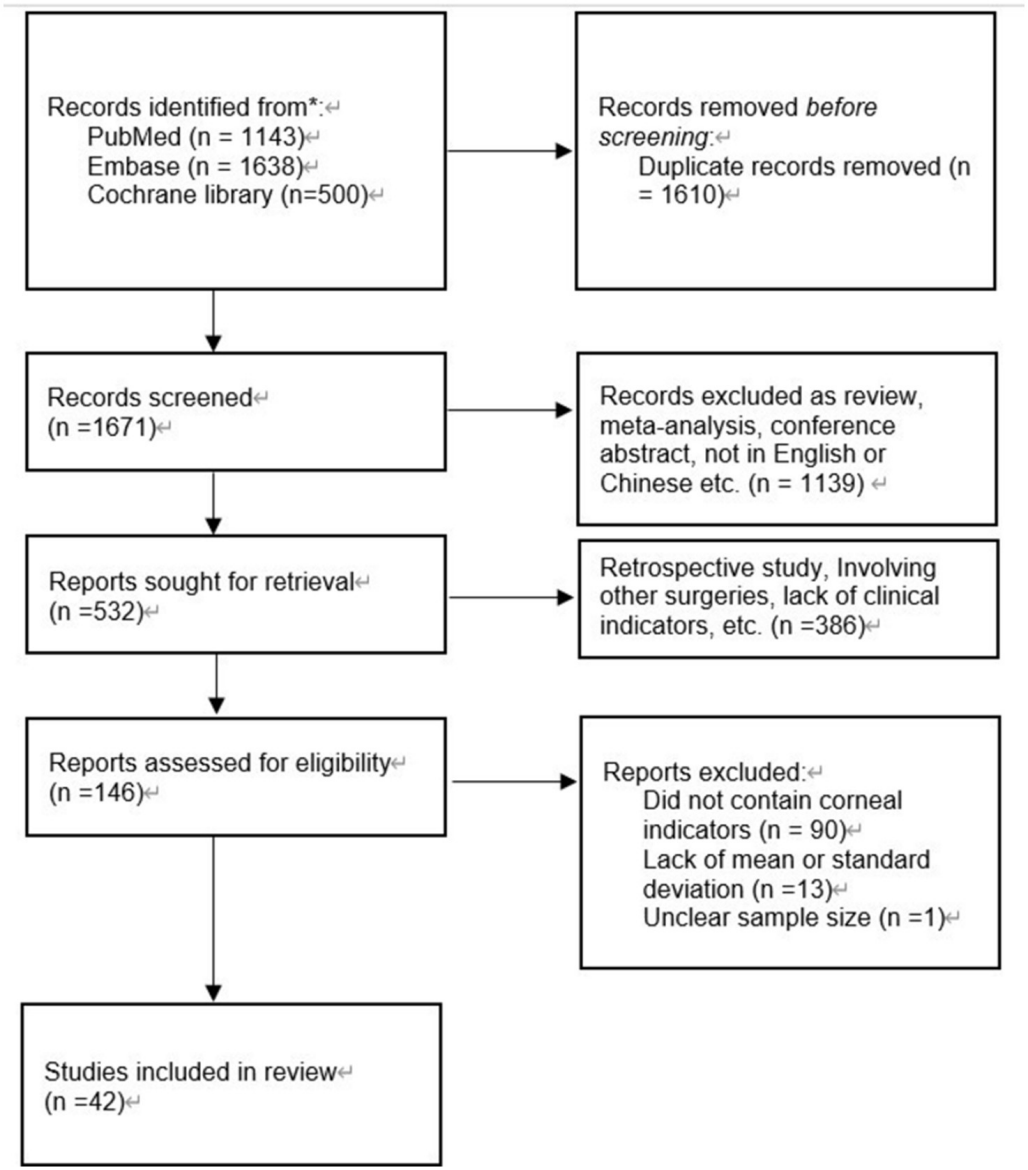
Flow diagram for identification of relevant studies on the corneal impact of FLACS and CPS.

A total of 3281 studies were identified originally. One thousand six hundred ten duplicates were discarded. One thousand six hundred seventy-one left studies were screened by title and abstract. A full-text examination was conducted when necessary. After excluding all research that did not meet the criteria, 42 [26–67] trials remained (Fig 1). Of the included studies, 23 were RCTs, and 19 were comparative cohort studies. Totally 3916 eyes underwent FLACS, and 3736 eyes underwent CPS. Characteristics of all the trials are recorded in Table 1. The quality assessment of RCTs is presented in S1 and S2 Figs, while that of the comparative cohort is in S1 Table.

**Table 1:** Characteristics of the included studies.

author	year	study design	FLACS platform	country	age (mean±sd)		sex (male: female)		no. of eye		follow-up
					FLACS	CPS	FLACS	CPS	FLACS	CPS	
Abell	2014	cohort	Catalys	Australia	72.4±10.1	72.6±9.8	135:270	NA	405	215	6m
Abell	2013	cohort	Catalys	Australia	72.8±10.5	71.8±10.8	69:81	23:28	150	51	3w
Al-Mohtaseb	2017	cohort	LenSx&catalys	USA	66.69±8.64	69.51±8.13	26:34	29:31	60	60	1m
Bascaran	2018	RCT	Victus	Spain	70.44±6.86		36:56		92	92	6m
Cavallini	2019	cohort	LDV Z8	Italy	75.45±7.88	75.06±9.32	19:30	16:41	80	80	3m
Chee	2021	RCT	Victus	Singapore	72.3±9.5	75.8±8.0	24:21	27:21	45	48	1m
Chen	2017	cohort	LenSx	China	68.38±8.45	70.27±8.53	18:29	21:27	47	48	3m
Chlasta-Twardzik	2019	RCT	LDV Z8	Pland	79.08±5.51	74.59±8.10	6:20	18:43	26	61	6m
Conrad-Hengerer	2013	RCT	Catalys	Germany	70.9		27:46		73	73	3m
Day	2021	RCT	LDV Z8	UK	68±10		48:52		392	393	12m
Day	2020	RCT	LDV Z8	UK	68±10	68±10	182:210	192:201	392	393	3m
Day a	2020	RCT	LDV Z8	UK	NA	NA	NA	NA	292	311	12m
Duan	2017	cohort	LenSx	China	NA	NA	NA	NA	74	74	3m
Dzhaber	2020	RCT	LenSx	USA	68.3±9.1		NA	NA	67	67	3m
Fan	2018	RCT	LenSx	China	66.1±9.2	63.9±12.5	3:7	2:6	16	15	12m
Gao	2018	cohort	NA	China	66.32±6.12	65.12±7.15	28:31	25:22	59	47	3m
Hansen	2020	RCT	LenSx	USA	68.7±8.5	69.0±14.1	27:44	25:39	64	71	3m
Kanellopoulos	2016	cohort	LenSx	Greece	67.3±11.99	69.92±11.73	27:40	29:37	67	66	12m
Kelkar	2020	cohort	Catalys	India	64.5±9.7	65.4±8.4	56:33	57:41	89	98	6m
Khan	2017	RCT	LenSX	Pakistan	NA	NA	23:25		25	25	1m
Krarup	2019	RCT	LensAR	Denmark	NA	NA	52:56		81	81	6m
Krarup	2021	RCT	LensAR	Denmark	75		17:17		31	31	6m
Krarup	2014	cohort	LensAR	Denmark	NA	NA	NA	NA	47	47	3m
Liu	2016	cohort	NA	China	50.1±3.3	49.6±2.6	15:6	14:7	21	21	12m
Liu	2021	RCT	LDV Z8	Singapore	69.5±6.9		48:37		78	78	12m
Mencucci	2020	cohort	LenSx	Italy	73.9±7.7	74.5±5.8	NA	NA	20	20	6m
Mursch-Edlmayr	2017	RCT	Victus	Germany	72±6		31:19		50	50	6m
Niu	2018	cohort	LenSx	China	67.12±5.64	66.39±5.23	32:38	35:47	107	126	3m
Oka	2021	RCT	LenSx	Japan	73.4±6.5		20:33		53	53	7m
Pisciotta	2018	cohort	LDV Z8	Italy	74.07±8.48	75.72±9.16	10:20	8:22	30	30	3m
Ranjini	2017	cohort	LenSx	India	NA	NA	NA	NA	55	55	1m
Reddy	2021	cohort	Catalys	India	59.5±9.5	58.25±10.1	11:9	22:18	20	40	5w
Roberts	2019	RCT	LenSx	UK	69.9±10.9	70.5±9.8	100:100	82:118	200	200	1m
Schargus	2015	RCT	Catalys	Germany	71.8		15:22		37	37	6m
Schroeter	2021	RCT	LenSx	Switzerland	70.5±8.3	69.6±8.1	31:34	27:38	65	65	3m
Shi	2020	RCT	LenSx	China	61.09±10.87		144:134		150	150	3m
Takacs	2012	RCT	LenSx	Hungary	65.81±12.42	66.93±10.99	10:28	15:23	38	38	1m
Vasavada	2019	RCT	LenSx	India	67.21±11.11	63.70±11.84	NA	NA	91	91	6m
Wu	2017	RCT	NA	China	62.9±4.8	61.7±5.2	NA	NA	85	105	3m
Yang	2019	cohort	LenSx	China	60.51±3.41	61.43±3.46	25:22	24:23	47	47	3m
Yu	2015	cohort	LensAR	China	62.3±11.6	56.5±16.6	NA	NA	25	29	3m
Yu	2016	cohort	LenSx	China	69.66±9.27	72.74±8.83	33:37	23:31	70	54	6m

### Endothelial cell loss rate (ECL%)

**Fig 2:**
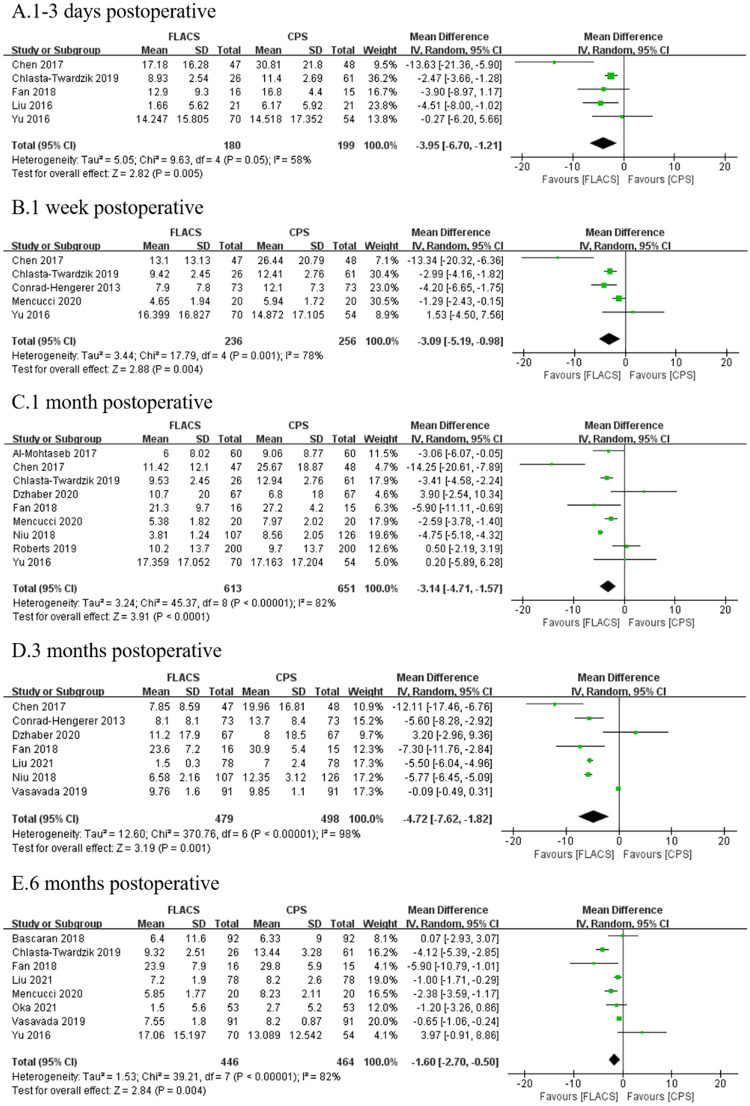
Forest plot of postoperative ECL% between FLACS and CPS at A. 1–3 days, B. 1 week, C. 1 month, D. 3 months, and E. 6 months.

Fifteen studies reported postoperative ECL%. FLACS group demonstrated significantly lower ECL% at 1–3 days (WMD: -3.95, 95%CI: -6.70, -1.21, P = 0.005), 1 week (WMD: -3.09, 95%CI: -5.19, -0.98, P = 0.004), 1 month (WMD: -3.14, 95%CI: -4.17, -1.57, P<0.0001), 3 months (WMD: -4.72, 95%CI: -7.62, -1.82, P = 0.001) and 6 months (WMD: -1.60, 95%CI: -2.70, -0.50, P = 0.004) postoperatively (Fig 2).

### Endothelial cell density (ECD) and Endothelial cell loss (ECL)

**Fig 3:**
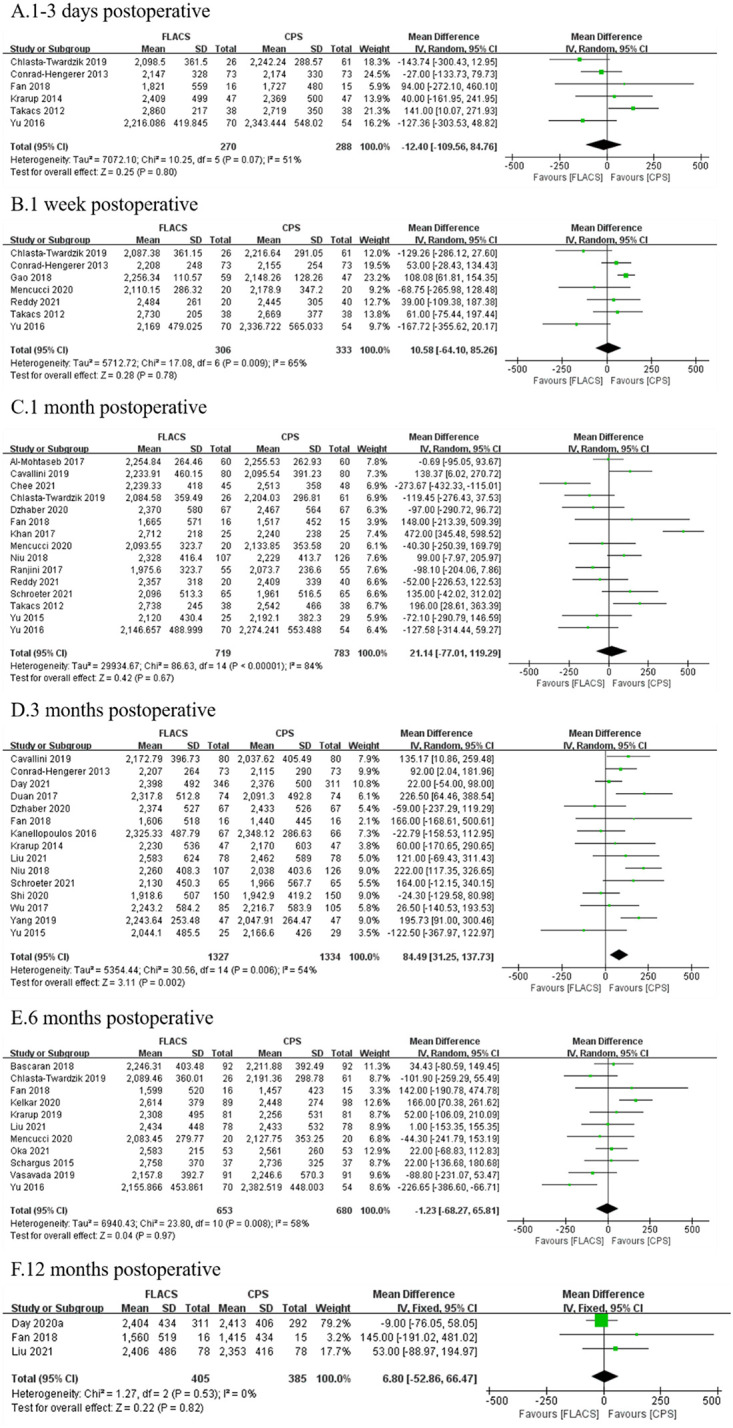
Forest plot of postoperative ECD between FLACS and CPS at A. 1–3 days, B. 1 week, C. 1 month, D. 3 months, E. 6 months, and F. 12 months.

There was no significant difference in ECD at 1–3 days (WMD:-12.40, 95%CI:-109.56, 84.76, P = 0.80), 1 week (WMD:10.58, 95%CI:-64.10, 85.26, P = 0.78), 1 month (WMD:21.14, 95%CI:-77.01, 119.29, P = 0.67), 6 months (WMD:-1.23, 95%CI:-68.27, 65.81, P = 0.97) and 12 months (WMD:6.80, 95%CI:-52.86, 66.47, P = 0.82) after surgery between two groups, and significant difference at 3 months (WMD: 84.49, 95%CI:31.25, 137.73, P = 0.002, Fig 3).

**Fig 4:**
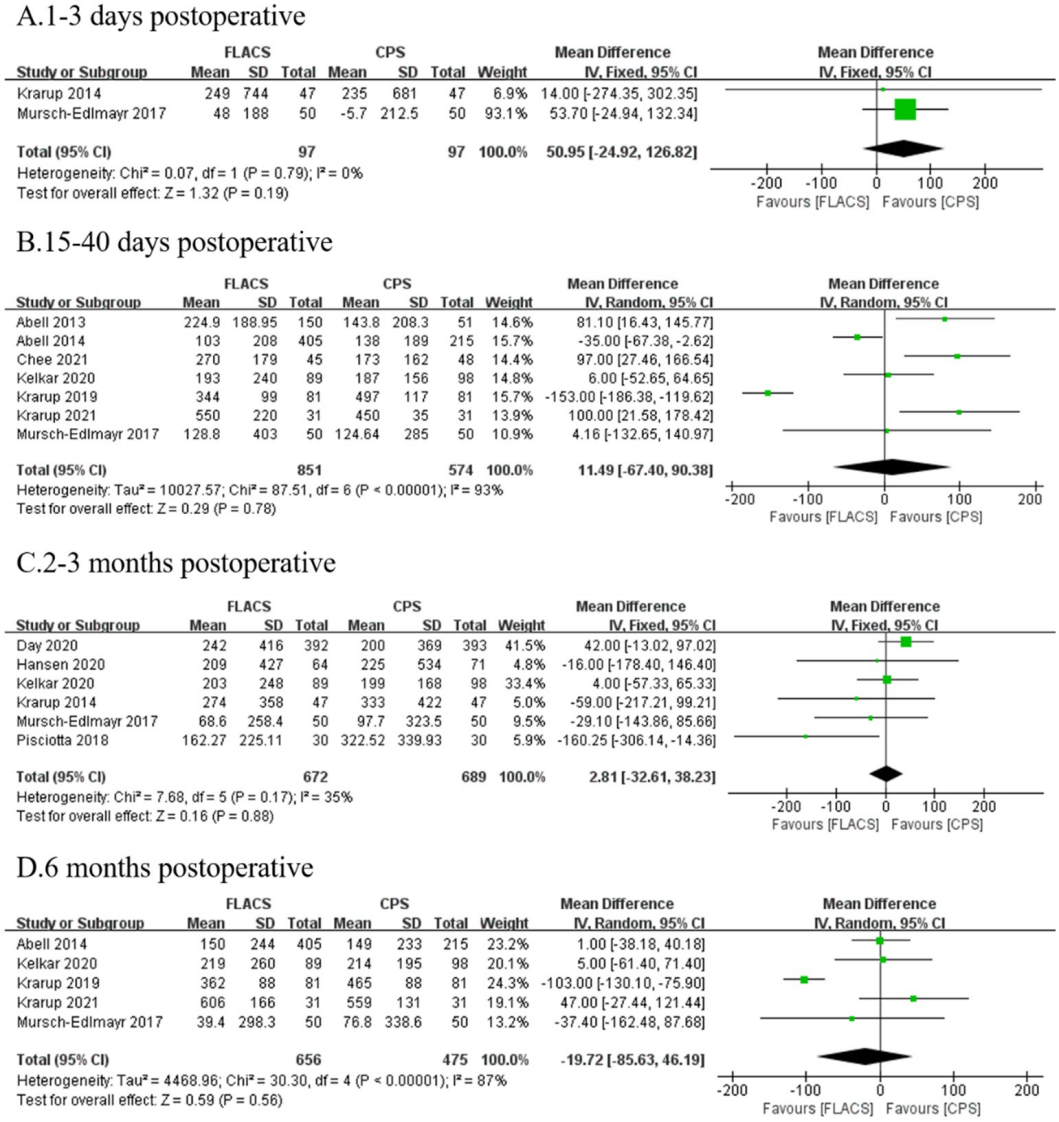
Forest plot of postoperative ECL between FLACS and CPS at A. 1–3 days, B. 15–40 days, C. 2–3 months, and D. 6 months.

Similarly, there was no significant difference in ECL at 1–3 days (WMD: 50.95, 95%CI: -24.92, 126.82, P = 0.19), 15–40 days (WMD: 11.49, 95%CI: -67.40, 90.38, P = 0.78), 2–3 months (WMD:2.81, 95%CI: -32.61, 38.23, P = 0.88), and 6 months (WMD: -19.72, 95%CI: -85.63, 46.19, P = 0.56) after surgery between two groups (Fig 4).

### Central corneal thickness (CCT)

**Fig 5:**
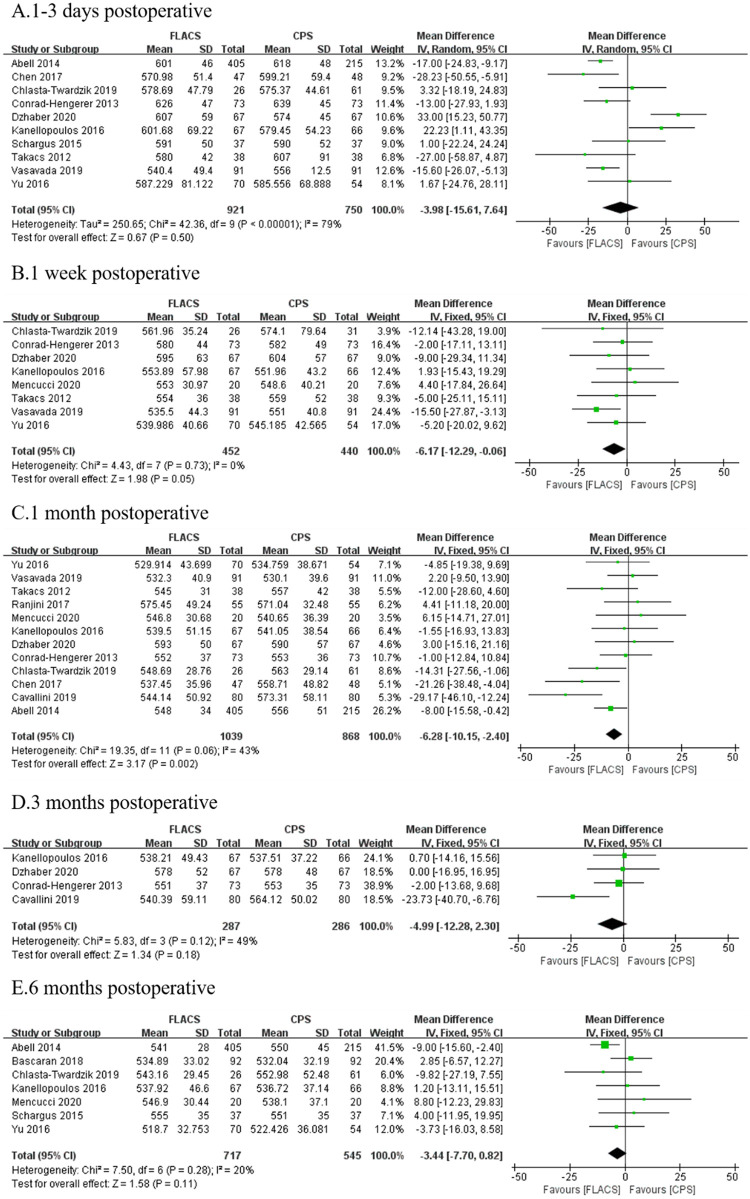
Forest plot of postoperative CCT between FLACS and CPS at A. 1–3 days, B. 1 week, C. 1 month, D. 3 months, and E. 6 months.

Fifteen studies reported postoperative CCT. No statistically significant difference was found between FLACS and CPS at 1–3 days (WMD: -3.98, 95%CI: -15.61, 7.64, P = 0.50) after surgery. Significantly lower CCT was observed in FLACS compared to CPS at 1 week (WMD: -6.17, 95%CI: -12.29, -0.06, P = 0.05) and 1 month (WMD: -6.86, 95%CI: -10.15, -2.04, P = 0.002). Whereas, later at 3 months (WMD: -4.99, 95%CI: -12.28, 2.30, P = 0.18) and 6 months (WMD: -3.44, 95%CI: -7.70, -0.82, P = 0.11), there was no statistically significant difference between two groups (Fig 5).

### Percentage of hexagonal cells (6A)

**Fig 6:**
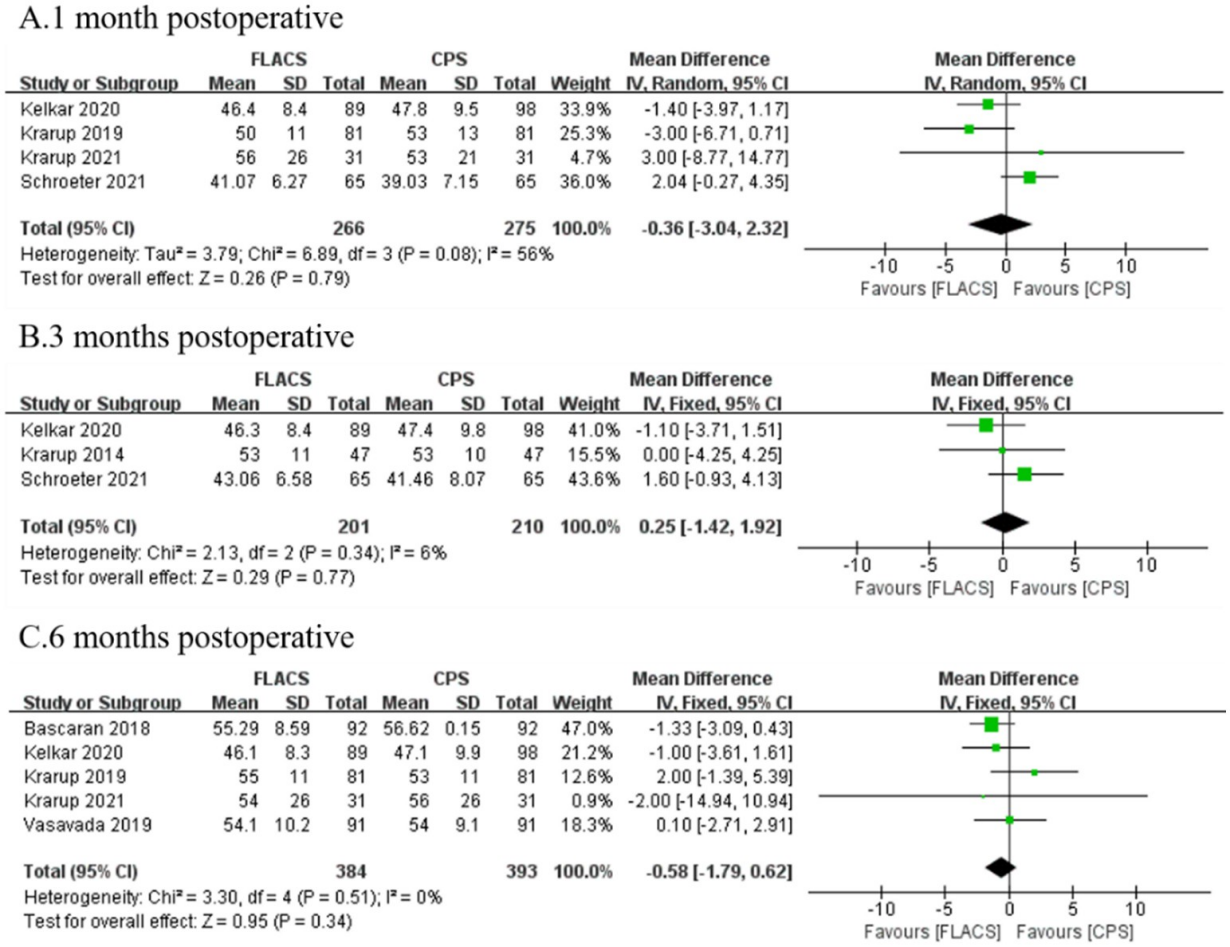
Forest plot of postoperative 6A between FLACS and CPS at A. 1 month, B. 3 months, and C. 6 months.

As was reported by seven researches, no significant difference was found between FLACS and CPS group at 1 month (WMD: -0.36, 95%CI: -3.04, 2.32, P = 0.79), 3 months (WMD: 0.25, 95%CI: -1.42, 1.92, P = 0.77) and 6 months (WMD: -0.58, 95%CI: -1.79, -0.62, P = 0.34, Fig 6).

### Coefficient of variance (CoV)

**Fig 7:**
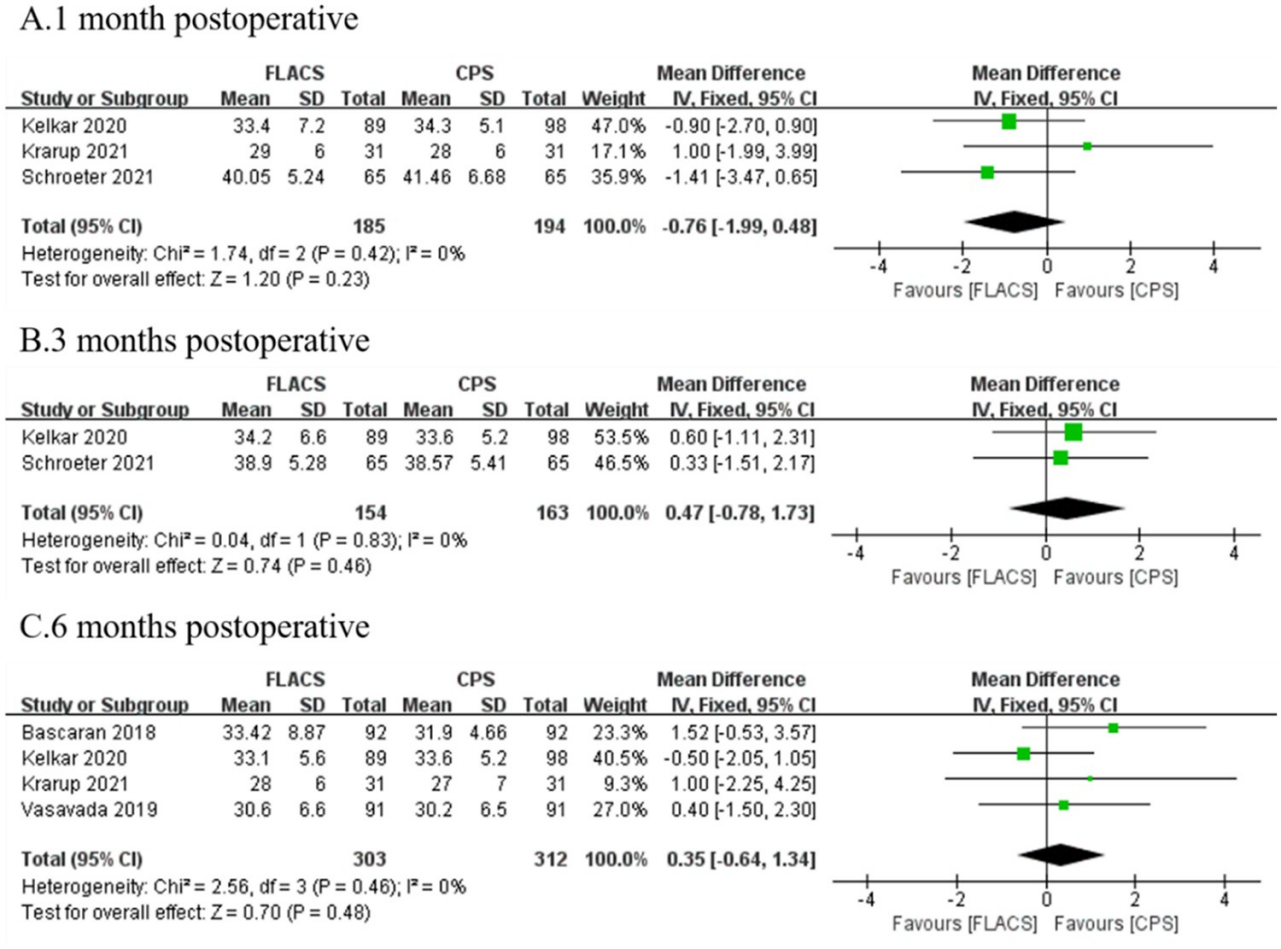
Forest plot of postoperative 6A between FLACS and CPS at A. 1 month, B. 3 months, and C. 6 months.

Five researches reported CoV were enrolled. No significant difference was found between the two groups at 1 month (WMD: -0.76, 95%CI: -1.99, 0.48, P = 0.23), 3 months (WMD: 0.47, 95%CI: -0.78, 1.73, P = 0.46) and 6 months (WMD: 0.35, 95%CI: -0.64, 1.34, P = 0.48, Fig 7).

## Discussion

This meta-analysis study showed the impact of FLACS on postoperative corneal endothelial injury compared to CPS. FLACS reduced ECL% significantly at each time point postoperatively. And CCT favored FLACS at 1 week and 1 month early after surgery.

The ECL% of the FLACS group was significantly lower than that of the CPS group at each time point during 6 months postoperatively, indicating that FLACS has reduces the injury of corneal endothelial cells. This was consistent with previously published meta-analysis [22]. Cataract surgical injury may result in a decrease in corneal endothelial cells, thus affecting the function of the cornea, and leading to corneal edema. Several factors had been reported to be involved in endothelial cell loss, such as ultrasound energy, phacoemulsification time, irrigation time, and usage of balanced salt solution during operation [68–70]. In FLACS, the nucleus of the lens is pre-fragmented by a femtosecond laser instead of manipulation. This allows less application of ultrasound energy and irrigating solution during cataract surgery [29], thereby reducing its damage to endothelial cells. As was reported by Abell [27] and Oshika [71], the effect of FLACS may be due to the lower requirement of effective phacoemulsification time (EPT), ultrasound energy, and irrigation fluid compared to CPS.

Lacking regenerative ability, the total amount of cells was no longer compensated after endothelial cell loss. Instead, migration and enlargement of residual cells occur as a corneal repairing procedure [9]. The remaining cells migrate to the injured area, resulting in a gradual increase in endothelial cell density when measuring at the center of the cornea [12]. This process takes months [72], therefore, lower ECL% on the first day after surgery indicated less damage to the corneal endothelium in the FLACS group. The long-term follow-up (3 months, 6 months) in the general population subgroup (S3 Fig) showed no significant difference between the two groups. It meant that the corneal endothelium can be repaired to a similar level in the FLACS and CPS groups. And the low ECL% in the FLACS group at 1 week and 1 month after surgery indicated that the corneal endothelial repair was faster in the FLACS group.

Differences in ECL% at 3 and 6 months after surgery proposed a persistent effect of FLACS and CPS on the cornea, but we found that the main impact may come from studies targeted to a specific population. Subgroup analysis of the general population revealed that ECL% at 1–3 days, 1 week, and 1 month in the FLACS group was still significantly lower than the CPS group, while no significant difference was found at 3 months and 6 months after surgery. Fuch’s syndrome or hard nuclear patients were not included in the subgroup. This result suggested that differences in ECL% in long-term follow-up are mainly caused by special populations. Fuchs’ endothelial corneal dystrophy patients were in a state of corneal decompensation preoperatively, with extremely low basal endothelial cell count [73]. In Fan’s study, the ECL% remained a significant difference until the endpoint of follow-up (12 months) [39]. And the mean ECL% in the CPS group (32.2% at 12 months) was also much higher than 4%-25%, which was previously reported in the general population [15]. Additionally, patients with hard nuclear may suffer higher ultrasound energy and prolonged phacoemulsification time to manifest the dens cataract; thus, intraoperative endothelial cells injury was even more severe [32]. In the general population, the ECL% was similar in the long-term follow-up in FLACS and CPS groups. It implied that the long-term effect of FLACS and CPS are comparable. As for the population with dysfunctional cornea, injury caused by surgery will persist [39]. Thus, we strongly recommend that FLACS may be the superior option for corneal dysfunction and hard nuclear patients.

Contrary to previously published meta-analysis [20, 21], ECD and ECL did not show significant differences across periods. It was possibly because that the absolute value of endothelial cells may be affected by the baseline level (preoperative ECD), while the ECL% can rule out the influence. ECL% is the percentage of endothelial cell loss in the preoperative endothelial cell density, calculated by the formula: ECL% = ((preoperative ECD- postoperative ECD)/preoperative ECD) *100%. Compared with ECD and ECL, ECL% appears to be less affected by interference factors. For example, Al-Mohtaseb [28] reported that baseline ECD in the FLACS group (2,408.78 ± 169.73) was significantly lower than that of the CPS group (2,486.29 ± 154.37, P = 0.03). Postoperatively, there was no difference in ECD between the two groups (FLACS: 2,254.84 ± 264.46, CPS: 2,255.53 ± 262.93, P = 0.49). However, ECL% favored the FLACS group (P = 0.04). Although the postoperative ECD was the same, there were differences in the number of endothelial cells loss between the two groups; thus, the ECD may not be an accurate reflection of endothelial cell change. The same is true for ECL, where the same number of ECL accounts for different ratios when the two groups are at different baselines [42]. Given these conditions, ECL% might be a more objective indicator to represent endothelial cell changes because the preoperative variance in ECD was removed.

In addition, different surgical approaches, such as manual or femtosecond-assist corneal incision, can also influence the endothelium. Femtosecond incision was thought to cause further damage to the cornea [74]. Femtosecond laser acts on the capsular bag when pretreating cataracts. While in the step of laser-assisted corneal incision, the laser energy directly conveys to the corneal endothelium [26]. Furthermore, microbubbles arising from laser-induced corneal rupture can influence the surface tension of endothelium and amplify the damage to it [75]. However, some studies lacked a description of this step; thus, it was difficult for us to perform a subgroup analysis of corneal incisions.

Across all time points, ECD at 3 months after surgery differed from the others. We noticed studies that recorded corneal data only at 3 months after surgery [60, 67], which might exert a large impact on the 3 months postoperative outcomes of ECD. Although the ECD in the FLACS group was significantly higher than that in the CPS group, the difference disappeared when only RCTs were included (S4 Fig). Meanwhile, the heterogeneity in the RCT subgroup decreased significantly (I^2^ = 4%). RCT studies reduce bias due to randomized grouping and are considered more reliable than cohort studies. We, therefore, supposed that the subgroup results of the RCT were more convincing at this time point. At other time points, there was no significant difference in ECD between the two groups when considering the RCT studies only (S4 Fig).

CCT represents the degree of corneal edema and is also an evaluation index of corneal endothelial function [76]. Our study demonstrated no significant difference in CCT 1–3 days after FLACS and CPS, which indicated similar corneal edema caused by two types of cataract surgery. This was in contrast to earlier meta-analyses. However, we performed a subgroup analysis of the femtosecond platform and found that in studies using the Catalys (Johnson & Johnson Vision Care, Inc.) femtosecond platform, CCT was significantly smaller in the FLACS group (S5 Fig). It may be caused by different docking modes. In Catalys femtosecond platform, a liquid optical immersion interface (LOI) [77] is applied to the docking phase. Correspondingly, LenSx platforms use curved contact lens interface [78]. Since LOI does not directly compress the cornea, it exerts less pressure, and consequently, induces less damage to the shape of the cornea [79]. Because of the fact above, we supposed Catalys performs a better protective effect on corneal endothelium since there is no direct contact with the cornea during the femtosecond-laser period. Currently, there was no study proposing that Catalys laser platform has better endothelium protection than LenSx. And subgroup analyses of platforms at more time postoperatively had no significant results due to the limited number of studies across different platforms. We look forward to more studies on the corneal effects of femtosecond platforms in the future. CCT was significantly smaller than CPS at 1 week and 1 month in the FLACS group, while there was no significant difference at 3 months and 6 months. This implied that corneal edema resolved faster in the FLACS group, and the long-term effects of FLACS and CPS on corneal edema were similar.

CoV and 6A demonstrated that neither proved significant differences at any time point, indicating a similar morphological change of endothelial cells after surgery. Alteration in cell shape and size occurs during corneal repairment, and these two indicators represent endothelial functional capacity. It suggested that there was no difference in the effect of the two surgeries on the function of the residual endothelial cells. Interestingly, a subgroup analysis of Schroeter’s study [55] showed that CoV decreased when EPT lessened. Although the results were highly consistent, the number of studies and the follow-up time points on these two indicators were not rich enough. Therefore, more follow-up articles and subgroup analyses are necessary.

In addition, we should also note that some reports have followed up on the long-term effects of FLACS and CPS for one year. The results showed that there was no significant difference in a long-term vision, complications, and corneal effects between the two groups [36, 80]. It is worth noting that FLACS has a higher economic cost, which may also be a problem to be considered when selecting surgical methods [81].

Unavoidably, there were limitations to this meta-analysis. Firstly, the postoperative follow-up time was only 6 months, which was due to insufficient follow-up data beyond 6 months. We look forward to more long-term follow-up articles. Secondly, the included studies were from different regions, using different CPS platforms, implemented by doctors of varying proficiency, making it difficult to unify patients’ preoperative baselines, resulting in increased heterogeneity. This meta-analysis was restricted to data from published studies, so information bias could not be fully ruled out if studies with small sample-size or unpublished data exist. And we only included clinical studies published in Chinese and English, which may lead to language bias.

## Conclusions

In conclusion, FLACS reduced corneal injury in the early postoperative period. Early postoperative corneal edema recovered faster than CPS. For patients with fewer endothelial cells, it is strongly recommended to consider FLACS first.

## Supporting information

S1 Fig Risk of bias graph: Based on researchers’ opinions about each risk of bias item, percentages are presented for all included studies. (TIF)

S2 Fig Assessment of the risk of bias in the included RCTs. Green circle (+): Low risk, Red circle (−): High risk,?: Unclear.
(TIF)

S3 Fig Forest plot of postoperative ECL% in general population between FLACS and CPS at A. 1–3 days, B. 1 week, C. 1 month, D. 3 months, and E. 6 months. (TIF)

S4 Fig Forest plot of postoperative ECD between FLACS and CPS that only included RCTs at A. 1–3 days, B. 1 week, C. 1 month, D. 3 months, and E. 6 months. (TIF)

S5 Fig Subgroup analysis of postoperative CCT between FLACS and CPS at 1–3 days. (TIF)

S1 Table The Newcastle-Ottawa Scale (NOS) of cohort studies. (PDF)

S1 Checklist PRISMA 2020 checklist. (DOCX)
