# Supplementary figures and images for: Comparison of femtosecond laser-assisted cataract surgery and conventional phacoemulsification on corneal impact: A meta-analysis and systematic review
Source: PLoS One. 2023 Apr 14;18(4):e0284181. doi: 10.1371/journal.pone.0284181 (PMC10104330; doi:10.1371/journal.pone.0284181)

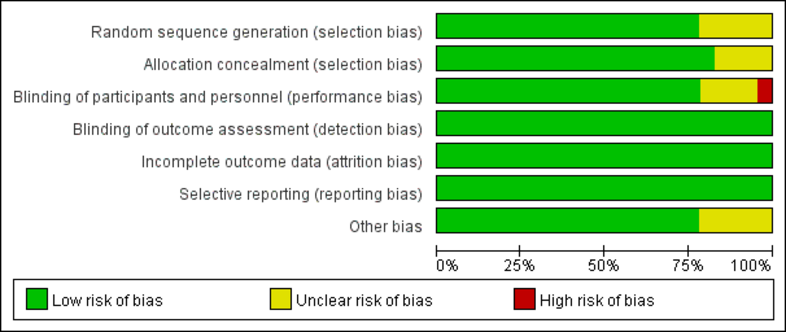

Supplement: S1 Fig — (TIF) [file pone.0284181.s001.tif]

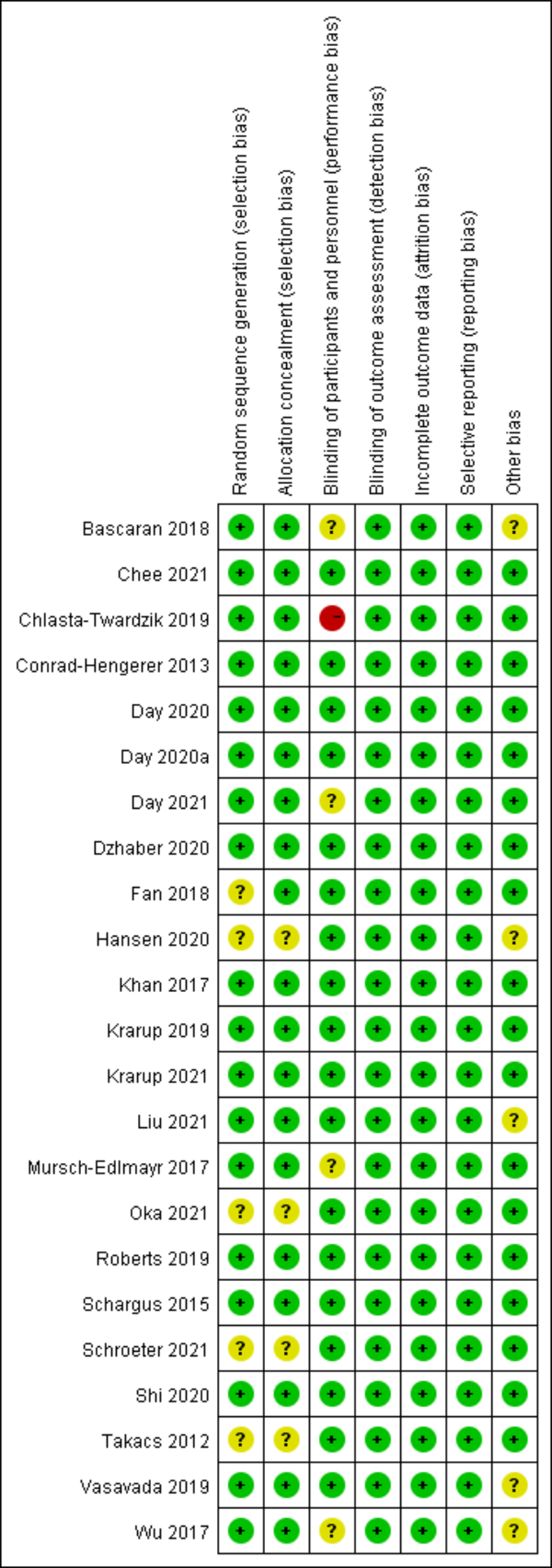

Supplement: S2 Fig — Green circle (+): Low risk, Red circle (−): High risk,?: Unclear. (TIF) [file pone.0284181.s002.tif]

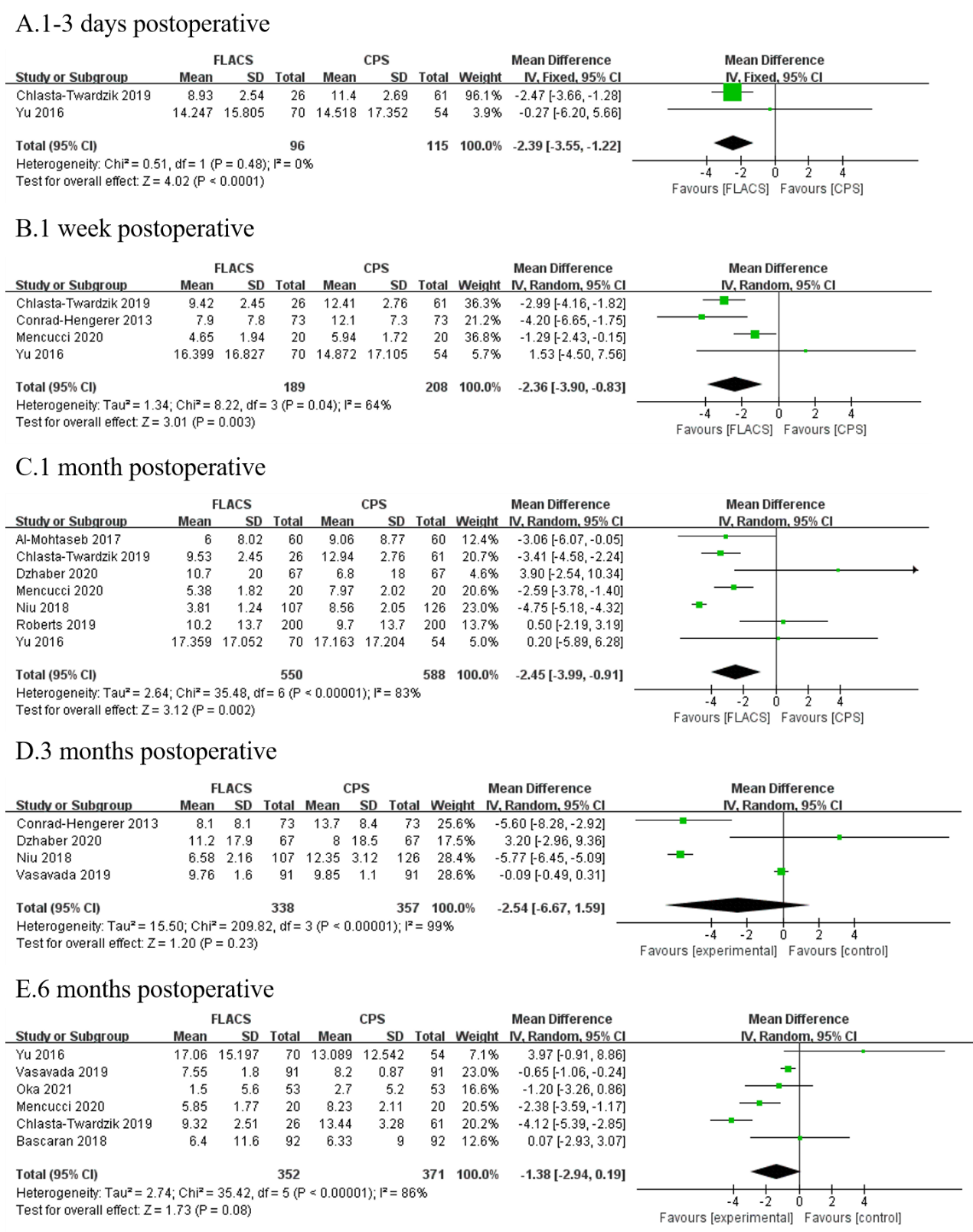

Supplement: S3 Fig — (TIF) [file pone.0284181.s003.tif]

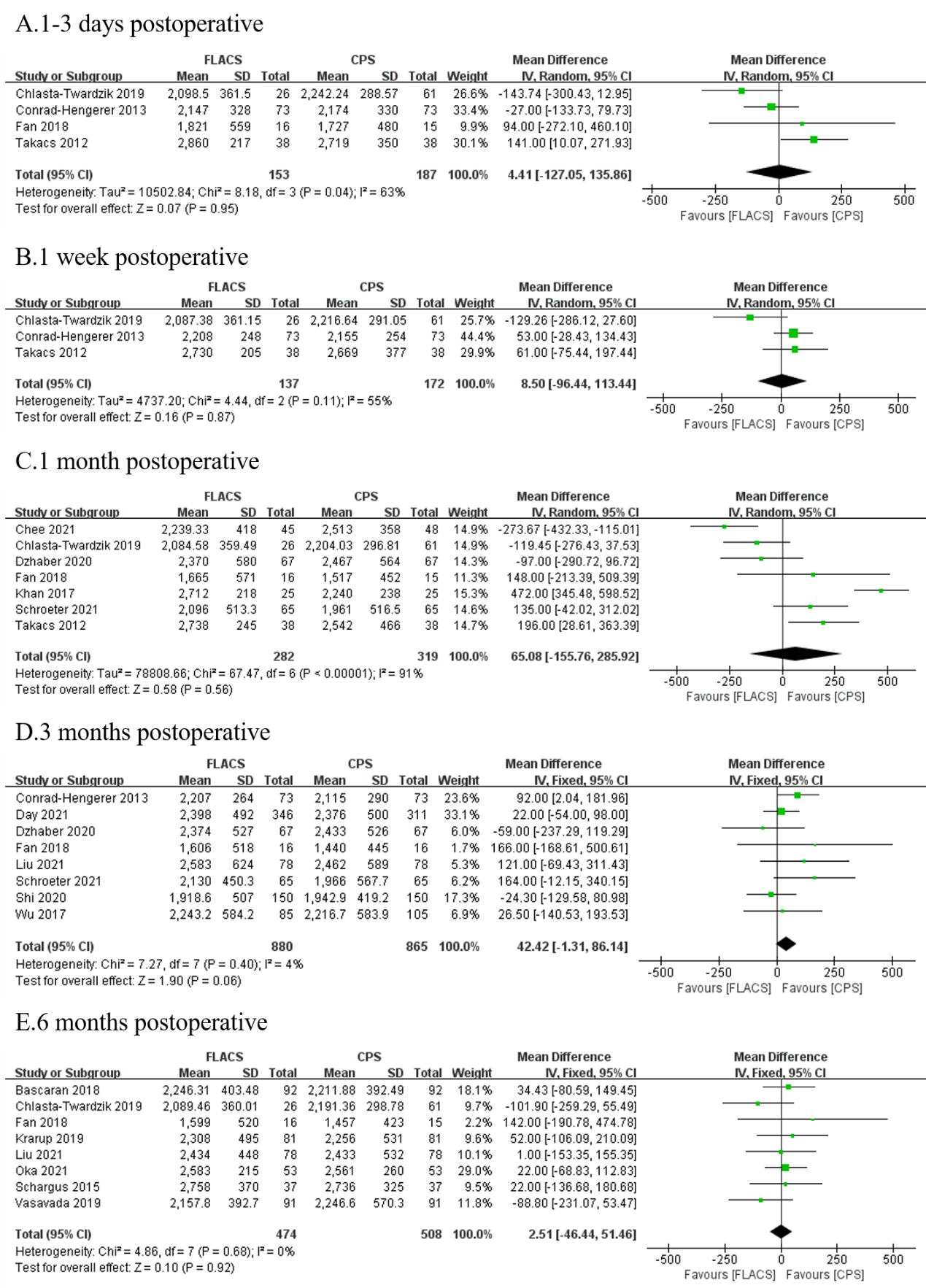

Supplement: S4 Fig — (TIF) [file pone.0284181.s004.tif]

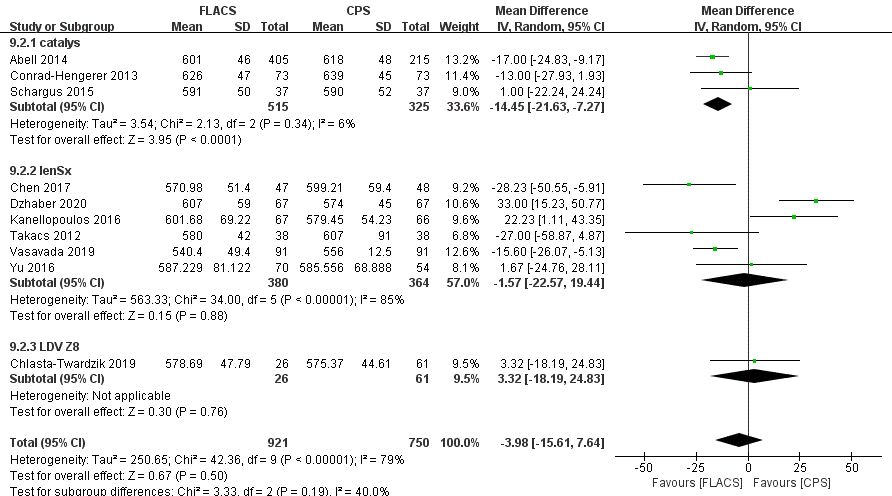

Supplement: S5 Fig — (TIF) [file pone.0284181.s005.tif]
