# Supplementary material for: Comparison of femtosecond laser-assisted cataract surgery and conventional phacoemulsification on corneal impact: A meta-analysis and systematic review
Source: PLoS One. 2023 Apr 14;18(4):e0284181. doi: 10.1371/journal.pone.0284181 (PMC10104330; doi:10.1371/journal.pone.0284181)
Supplement: S1 Table — (PDF) [file pone.0284181.s006.pdf]

author	year	country	Selection	Comparability	Outcome	Total score
Abell	2014	Australia	****	*	***	********
Abell	2013	Australia	****	*	***	********
Al-Mohtaseb	2017	USA	****	*	***	********
Cavallini	2019	Italy	****	*	***	********
Chen	2017	China	****	*	***	********
Kanellopoulos	2016	Greece	****	*	***	********
Kelkar	2020	India	****	*	***	********
Krarup	2014	Denmark	****	*	***	********
Mencucci	2020	Italy	****	*	***	********
Pisciotta	2018	Italy	****	*	***	********
Ranjini	2017	India	****	*	***	********
Reddy	2021	India	****	*	***	********
Yu	2015	China	****	*	***	********
Yu	2016	China	****	*	***	********
Duan	2017	China	****	*	***	********
Gao	2018	China	****	*	***	********
Liu	2016	China	****	*	***	********
Niu	2018	China	****	*	***	********
Yang	2019	China	****	*	***	********

S3 Table. The Newcastle-Ottawa Scale (NOS) of cohort studies.
